# Social Capital and Depression Among Adolescents Relocated for Poverty Alleviation: The Mediating Effect of Life Satisfaction

**DOI:** 10.3390/healthcare13070743

**Published:** 2025-03-27

**Authors:** Dan Guo, Le Yang, Li Wang, Qi Yu

**Affiliations:** 1 School of Management, Shanxi Medical University, Taiyuan 030000, China; guodan@sxmu.edu.cn (D.G.); yangle05@hotmail.com (L.Y.); 2 Center for Health Management and Policy Research, Shanxi Medical University, Taiyuan 030000, China; 3 School of Public Health, Shanxi Medical University, Taiyuan 030000, China; wangli_1@sxmu.edu.cn; 4 Big Data Laboratory for Clinical Decision Research, Shanxi Medical University, Taiyuan 030000, China

**Keywords:** social capital, depression, adolescents, life satisfaction, mediating effect

## Abstract

**Background:** China’s relocated for poverty alleviation policy has played a pivotal role in eradicating extreme poverty nationwide. However, adolescents relocating with their parents may face multifaceted challenges, including abrupt shifts in their living environments, the reconstruction of social capital, and the psychological turbulence inherent to adolescence. **Objectives**: We aimed to explore predictors of reducing depressive symptoms in relocated adolescents. We analyzed the associations between social capital, life satisfaction, and adolescent depression. **Methods**: This study investigated 631 adolescents aged 10–19 years from 24 relocation for poverty alleviation resettlement sites in Shanxi Province. Respondents completed basic demographic information and questionnaires on adolescent social capital, life satisfaction, and depressive symptoms. The mediating role of life satisfaction was assessed using PROCESS 3.4 analysis. **Results**: The mean social capital score of the adolescents was 31.96 ± 3.666, the mean life satisfaction score was 23.21 ± 6.282, the mean depression score was 4.03 ± 5.503, and the depression detection rate was 15.2%. We found that social capital was significantly positively correlated with life satisfaction (r = 0.363, *p* ˂ 0.05), both social capital and life satisfaction were negatively correlated with depressive symptoms (r = −0.362, *p* ˂ 0.05; r = −0.398, *p* ˂ 0.05), and life satisfaction partially mediated the association between social capital and depressive symptoms (mediating effect of 18.20%). **Conclusions**: Adolescents in communities relocated for poverty alleviation are overall satisfied with their lives, but some are experiencing some form of depression. Both social capital and life satisfaction are associated with lower depression levels, and those with higher life satisfaction are better able to cope with the changes in social capital associated with environmental changes after relocation, thus helping to reduce depressive symptoms.

## 1. Introduction

Adolescence is a vigorous yet vulnerable period of development characterized by rapid cognitive development and peak episodes of mental health disorders [1]. It is estimated that around 14.9–26.5% of adolescents worldwide suffer from depression [2,3]. This problem seems to be especially serious in China, where studies show that the prevalence of depression among Chinese adolescents is 24.3% [4]. Furthermore, the frequency and severity of these mental health disorders have increased among adolescents over the past two decades [5].

Poor mental health can have a significant impact on the health and development of adolescents and can lead to a variety of negative social consequences, such as alcohol and drug use, delinquent behavior, and dropping out of school [6]. Therefore, it is important to target key factors in order to alleviate mental health disorders in adolescents.

### 1.1. Conceptual Framework

According to the World Health Organization, mental health is characterized not only by the absence of mental health problems but also by the ability of an individual to “recognize his or her own capacity to cope with the normal stresses of life, to work productively, and to contribute to his or her community” [7]. Thus, in addition to addressing risk factors to reduce mental health problems, a strength-based view of psychology emphasizes the importance of identifying protective factors that promote self-esteem and life satisfaction in order to promote positive mental health and well-being [8].

The concept of “social capital”, developed by Bourdieu, has also been recognized as having a complex association with health [9]. According to Putnam et al., social capital refers to socially organized features such as trust, networks, and norms that can enhance social efficiency by facilitating coordinated action. Several studies have shown that social capital has a positive impact on self-assessed health [10,11,12,13]. And, social capital has been shown to be effective in reducing depressive symptoms in adolescents [14]. However, the mechanisms by which social capital influences adolescent depression need to be explored more.

Life satisfaction is an important aspect of subjective well-being, a subjective global judgment that people make about their lives as a whole [15]. Among the outcomes associated with adolescent depression, life satisfaction plays a major role [16]. For example, a representative population-based study of adolescents showed that life satisfaction was associated with positive social and health outcomes [17]. In addition, life satisfaction was found to be associated with depression and other mental health deficits [18].

### 1.2. Background of China’s Poverty Alleviation Policy

The United Nations Sustainable Development Goals (SDGs), particularly Goal 1 (“No Poverty”), aim to eradicate extreme poverty globally by 2030. China has played a transformative role in advancing this agenda through its innovative “Targeted Poverty Alleviation” strategy, which emphasized the precise identification of impoverished populations and tailored interventions, including relocation for poverty alleviation.

Relocation for poverty alleviation is an important means of poverty alleviation in China, in which poor people living in areas with poor ecological environments, poor natural conditions, high incidence of geological hazards, and other conditions that are not conducive to survival are relocated, in accordance with the principle of voluntariness and under the unified organization of the government, to areas with better infrastructure, employment, and public services. In the decade 2012–2022, at least 9.6 million people were lifted out of poverty through relocation for poverty alleviation. Among them, children and adolescents account for a significant proportion of the relocated population [19]. This approach has not only lifted China out of poverty, achieving the Sustainable Development Goals a decade ahead of their time, but has also set a benchmark for global poverty reduction.

The social capital of relocated adolescents may face a number of changes. First, the breakup and reconstruction of social networks: relocation may lead to the breakup of the original community relations, especially the loss of “bonding social capital” based on geographic location such as kinship and neighborhood, but at the same time, it may also promote the formation of “bridging social capital” through social interactions in the new community, such as cross-group ties and institutional support [20]. But, at the same time, the formation of “bridging social capital” may be facilitated through social interactions in the new community, such as cross-group ties and institutional support. Second, psychological adaptation and trust reconstruction: adolescents may face a crisis of trust when adapting to new environments, especially in culturally diverse neighborhoods, and require the intervention of external forces, such as the school and the community, in order to rebuild social trust and support networks [21,22].

As for the regions where relocation has been completed, the main task at this stage is to improve the social adaptability of the relocated population through the improvement of follow-up policies and to promote the comprehensive and healthy development of the relocated population.

Relocation for poverty alleviation may have complex effects on the mental health of migrant adolescents: on the one hand, relocation results in the breakup of the migrants’ original social relationships, the loss of existing resources, and the possibility of short-lived difficulties before new social relationships are established, thus affecting the physical and mental health of adolescents [23]; on the other hand, relocation can increase access to resources for those who were already in poverty, thus improving their quality of life and health resources.

The relocation of adolescents in poverty alleviation programs may influence their depressive symptoms through two primary pathways. Firstly, the transformation of social capital post-relocation—marked by direct improvements in housing, education, healthcare, and other living conditions—can enhance life satisfaction and subjective well-being, thereby mitigating depressive tendencies. Enhanced access to stable housing, quality schooling, and reliable medical services fosters a sense of security and optimism, which are critical psychological buffers against depression [24,25]. Secondly, the relocation enables parents to secure employment opportunities closer to home, increasing familial presence and cohesion. Concurrently, structured support systems from improved school environments and community management—such as mentorship programs, peer networks, and organized social activities—provide adolescents with emotional stability and practical assistance [26]. These combined factors—economic stability through parental employment, strengthened family bonds, and institutionalized social support—act synergistically to directly alleviate depressive symptoms by addressing both material deprivation and psychosocial stressors.

However, few studies have investigated the possible psychological mediators between adolescent social capital and depression, especially those that could help to enhance the positive effects of social capital.

### 1.3. Research Hypothesis

Based on the above analysis, this study proposes four hypotheses as follows:

**H1.**  *Social capital is positively correlated with life satisfaction.*

**H2.**  *Life satisfaction is negatively correlated with depressive symptoms.*

**H3.**  *Social capital is negatively correlated with depressive symptoms.*

**H4.**  *Life satisfaction mediates the association between social capital and depressive symptoms.*

Given the sensitivities of this group and the lack of studies using life satisfaction as a mediator of the relationship between social capital and depression, the present study aimed to measure the dimensions of life satisfaction, depression, and social capital in a representative sample of adolescents relocated for poverty alleviation. Evidence from this study may serve as a basis to help promote positive mental health and well-being among adolescents [6].

## 2. Materials and Methods

### 2.1. Data Sources

Shanxi Province is a key region in China’s implementation of the relocated for poverty alleviation policy. As the birthplace of Chinese civilization and a cradle of its historical and cultural heritage, the concept of social capital in Shanxi is inherently culturally embedded. Investigating the social capital of relocated adolescents under poverty alleviation programs and its impact on depression in this province holds significant representativeness, given its blend of cultural depth and socioeconomic diversity. Consequently, the findings can be generalized to most regions in China with similar social and economic development contexts.

In this study, a household survey was conducted in twenty-four relocation for poverty alleviation resettlement sites in eight counties of four cities (Taiyuan, Xinzhou, Lvliang, and Linfen) in Shanxi Province using multi-stage stratified sampling from June to August 2023.

We calculated the sample size using the formula $n=\frac{{Z_{a}}^{2}\times p(1-p)}{d^{2}\times p}$, where *n* is the number of participants in the study, the confidence level is taken as 95% (two-sided), and *Z* is the standard normal distribution bounds, *Z_a_* = 1.96. According to the 24.3% depression rate of Chinese adolescents in junior and senior high school in 2021 [4], *p* is taken as 24.3%, the allowable error *d* is taken as 10%, and, with a 20% nonresponse rate taken into account, the minimum sample size obtained from the calculations was 348 people. Based on the total number of adolescents in the sampled placements, 3480, the adolescents were sampled in each placement at a rate of 10–15%.

Questionnaires were administered to adolescents between the ages of 10 and 19, based on the World Health Organization’s definition of the adolescent age range [27], and a total of 631 valid questionnaires were collected. All study participants were informed about the study, and they voluntarily completed the survey.

Inclusion criteria: 1. adolescents aged 10–19 years living in one of the sampled pro-poor settlements; 2. have basic reading and communication skills; and 3. be willing to provide informed consent and participate in the study voluntarily. Exclusion criteria: 1. inability to understand the questionnaire questions or cognitive impairment; and 2. adolescents who do not live permanently in the settlement.

Before the survey was conducted, all investigators were given uniform training on the background of the project and relevant professional knowledge, and the purpose of the study and the instructions for completing the questionnaire were explained to them. During the formal survey, two investigators obtained the study participants’ informed consent and conducted a face-to-face questionnaire survey. At the end of the survey, the investigators checked the completeness of the questionnaire and collected it on the spot. On the same day, the questionnaires were rigorously reviewed to eliminate incomplete questionnaires, obvious logical errors, and other problems to ensure that the data were accurate and complete.

### 2.2. Variables

Depression Anxiety Stress Scale (DASS-21): In this study, we used the self-assessment scale developed by Lovibond et al., which is used to assess an individual’s negative emotional states within the week prior, thus measuring the severity of negative emotional symptoms [28]. The DASS-21 has 21 items across the three dimensions of depression, anxiety, and stress, with the items rated on a 4-point scale from 0 to 3 (0 = “does not apply”, 1 = “sometimes applies”, 2 = “often applies”, 3 = “always applies”). The final score is the total score multiplied by 2; the higher the score, the higher the severity of emotional states. Items 3, 5, 10, 13, 17, and 21 on the scale indicate depression-related conditions, with depression scores ≤ 9 being normal, 10–13 being mildly depressed, 14–20 being moderately depressed, 21–27 being severely depressed, and ≥28 being very severely depressed.

Social Capital Questionnaire for Adolescent Students (SCQ-AS): We used the SCQ-AS developed by Paiva et al. [29] to assess social capital. The questionnaire consists of 12 items with 3 levels of scoring, with items 9 and 10 being reverse-scored, and the total score is the sum of the scores of the 12 items. A higher total score indicates higher social capital. The questionnaire includes four dimensions: school cohesion (items 1, 2, 3, and 6), school friendship (items 4, 11, and 12), neighborhood social cohesion (items 7 and 8), and trust (items 5, 9, and 10).

Satisfaction with Life Scale (SWLS): This study used the SWLS developed by Diener et al. [30] to assess adolescents’ life satisfaction. The questionnaire consists of 5 items rated on a 7-point scale, excluding reverse scoring, and the total score is the sum of the scores of the 5 question items. The higher the total score, the higher the life satisfaction of the subject.

Control variables: With reference to the previous relevant literature [31,32], the control variables included in this study included basic demographic characteristics, mode of residence, whether the subjects were children, smoking, alcohol consumption, outdoor exercise, and other health-related behavioral conditions.

### 2.3. Statistical Analysis

**Figure 1:**
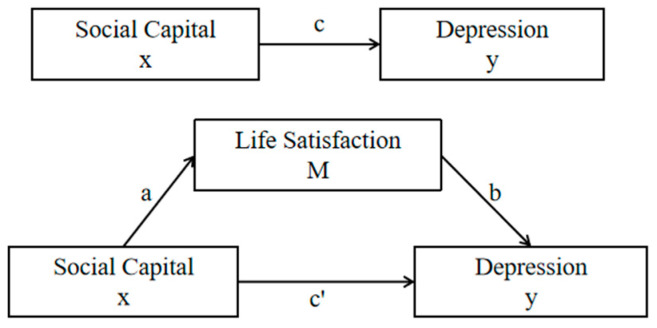
Hypothetical model for mediated effects analysis.

Data were analyzed using SPSS 25.0, with measures expressed as mean ± standard deviation ($\bar{x}$ ± S) and counts expressed as frequency and percentage. Pearson correlation was used to analyze the associations among social capital, life satisfaction, and depression. The mediating role of life satisfaction was assessed using PROCESS 3.4 analysis. The test procedure provided by Zhonglin et al. [33] was used to establish three regression variances for the independent variable (x), the mediating variable (M), and the dependent variable (y) in accordance with Figure 1, and the regression coefficients were tested sequentially in three steps. First, coefficient c was tested; if not significant, the mediation effect analysis was stopped. If coefficient c was significant, coefficient a and coefficient b were sequentially tested; if either a or b was not significant, a Sobel test should be performed. If both were also significant, we continued to test coefficient c′; the partial mediation effect of M is significant if c′ is also significant, and the full mediation effect of M is significant if c′ is not. In Figure 1, c is the total effect of x on y, and a × b is the mediating effect of M. The proportion of the mediating effect to the total effect was found using the formula a × b/c. This study performed 5000 bootstrap replicates and used them to determine 95% confidence intervals for indirect effects. The test level was α = 0.05.

## 3. Results

Respondents’ gender, age, only child status, parents’ education level, and health-related behaviors are shown in Table 1. Of the 631 adolescents, 96 (15.2%) were found to have symptoms of depression, of whom 46 (7.3%) were mildly depressed, 39 (6.2%) were moderately depressed, and 11 (1.7%) were severely/extremely depressed. The mean life satisfaction score of the interviewed adolescents was 23.21 ± 6.282, and the mean social capital score was 31.96 ± 3.666.

In order to test the hypothesis that life satisfaction plays a mediating role in the process by which social capital has an effect on depression, a bias-corrected nonparametric percentile bootstrap method was used to test for mediating effects using Model 4 of the PROCESS macro program in SPSS (Model 4 is a simple mediation model). Descriptive statistics and correlation analysis were first performed on the variables. The results showed that the total social capital score and all four of its dimensions showed a significant positive correlation with life satisfaction and a significant negative correlation with depression (Table 2).

The results of the mediation model indicated that social capital significantly positively predicted life satisfaction (β = 0.63, *p* < 0.001). When both social capital and life satisfaction were applied to the regression equation, both social capital (β = −0.44, *p* < 0.001) and life satisfaction (β = −0.16, *p* < 0.001) significantly negatively predicted depression levels. The four subdimensions of social cohesion, school friendships, neighborhood social cohesion, and trust also exhibited results consistent with the above two variables (Table 3).

**Figure 2:**
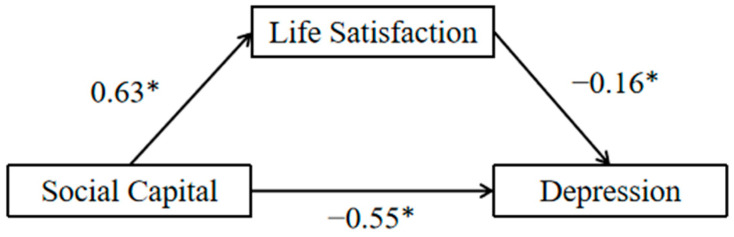
Path diagram of the mediating role of life satisfaction between social capital and depression. (Note: * *p* < 0.05).

The bootstrap 95% CI of the mediating effect did not contain 0 [0.06–0.15], indicating a significant mediating effect, and the mediating path is shown in Figure 2. Thus, life satisfaction plays a partial mediating role in the relationship between social capital and depression, and the mediating effect is 18.20% of the total effect.

In addition, the mediating effects of life satisfaction in the associations of school cohesion, school friendship, neighborhood social cohesion, and trust with depression were all significant, with mediating effects accounting for 21.37%, 17.67%, 21.79%, and 32.94% of the total effect, respectively (Table 4).

## 4. Discussion

The purpose of this study was to investigate the relationships among life satisfaction, social capital, and depressive symptoms in adolescents relocated for poverty alleviation and the mediating role of life satisfaction in the relationship between social capital and depressive symptoms. We tested the following four hypotheses: H1, social capital is positively correlated with life satisfaction; H2, life satisfaction is negatively correlated with depressive symptoms; H3, social capital is negatively correlated with depressive symptoms; and H4, life satisfaction mediates the association between social capital and depressive symptoms.

Consistent with H1, the results indicated a significant and moderate positive correlation between social capital and life satisfaction, a result that is consistent with the findings of existing studies [34,35]. The mean life satisfaction of adolescents was 23.21 ± 6.282, which was slightly higher than that in other relevant studies. The findings of Okwrji using the SWLS showed that 32.3% of adolescents aged 16–19 were dissatisfied with life [36,37]. Additionally, a four-year longitudinal study conducted by Shek [38] in Hong Kong showed that the mean life satisfaction based on the SWLS decreased from 19.38 ± 5.37 to 18.54 ± 5.27. Meanwhile, Moksnes and Espnes [39] showed that Norwegian girls had a mean life satisfaction score of 22.31 ± 6.01. In this study, based on the measurements of this instrument, the adolescents’ life satisfaction was above the neutral level, which meant that they were more satisfied with their lives.

The social capital scale used in this survey had four dimensions: school cohesion, school friendships, neighborhood social cohesion, and trust. Life satisfaction was significantly, albeit weakly to moderately, correlated with all types of social capital. The correlation between life satisfaction and school cohesion was the strongest. This suggests that a stronger sense of belonging and disciplinary climate at school are protective factors that influence adolescents’ life satisfaction. Meanwhile, according to related studies, both father–child communication and mother–child communication significantly and positively predicted the level of adolescent life satisfaction [40], whereas the lack of family social capital dimension in the SCQ-AS scale may underestimate the strength of the correlation between social capital and life satisfaction.

H2 was also confirmed in this study: adolescent life satisfaction was negatively associated with depressive symptoms. The detection rate of depression among adolescents relocated for poverty alleviation in the present study was 15.2%, and although it was low compared with the results of related studies [41,42], adolescent life satisfaction was significantly and moderately negatively correlated with depressive symptoms, a result that has been confirmed in the previous literature [43]. Therefore, the low depression among the adolescents in the sample of this study may be partially attributable to their high life satisfaction. Since self-consciousness such as sensitivity and goal identity increases during adolescence [44,45], life satisfaction during this period is an important psychological variable. Relevant studies have concluded that among the dimensions of life satisfaction, family satisfaction and self-fulfillment are the two dimensions that are most closely related to depressive symptoms [46]. In addition, adolescents’ life satisfaction has been found to significantly predict social (relationship with parents), behavioral (delinquency), and psychological (depression and anxiety) dimensions [6,47,48]. This suggests that life satisfaction is an important indicator of subjective well-being and can play a role in reducing the expression of depressive symptoms.

Furthermore, our study confirmed H3 (social capital was negatively associated with depressive symptoms), which is consistent with the findings of many studies [49]. Of the four dimensions of social capital, school cohesion and school friendship were the two dimensions with the highest degree of correlation. Adolescents can obtain resources and extend social support by participating in school activities and having a good relationship with teachers and peers. The sense of belonging and the disciplinary atmosphere of the school help adolescents to establish a basic normative perception of social integration, expanding children’s social capital and thus promoting the development of physical and mental health. Related studies have found that having a small number of close friends is a risk factor for depression in adolescents [50] and that social adversities such as bullying and social isolation also increase depression in adolescents [51]. Therefore, encouraging adolescents relocated for poverty alleviation to make a wide range of friends on campus and build partnerships and peer networks can help buffer the risk of depression.

In addition, neighborhood social cohesion and trust were also significantly negatively associated with depression, and although this association was weak, it was also consistent with many researchers’ findings. Neighborhood disadvantage has been shown to have a number of negative effects on adolescents, including a higher risk of depression [52]. Moreover, there is a cumulative effect of residential environment on adolescents; that is, adolescents living in disadvantaged neighborhoods for long periods of time have higher levels of depression and anxiety [53]. Therefore, it is important to provide better residential environments for adolescents to establish a stable social ecology for personal development.

Finally, the results confirmed H4, which was validated by a mediated effects model. The model verified the mediating effect of life satisfaction on the association between social capital (overall) and depression. The beta value of the test indicated a partial mediating role of life satisfaction in this association. These findings are consistent with the hypothesis that those who are more satisfied with their lives have better psychological states in the face of environmental change. Thus, while social capital is negatively associated with depressive symptoms, the analysis of these models suggests that being more satisfied with life may enhance this protective effect.

Life satisfaction in this study partially mediated the relationships between the dimensions of social capital and depression, with the mediators being, in descending order, school friendship, school cohesion, neighborhood social cohesion, and trust. This suggests that adolescents are in a network of partnerships, teacher–student relationships, and neighborhood relationships at school and in the community, all of which are resources that can help to improve overall subjective evaluations of life and thus reduce depressive symptoms. Existing research demonstrates that peers’ behavioral and mental health states significantly shape adolescents’ health-related behaviors (e.g., diet, physical activity, substance use), physical well-being, and psychological health [54,55,56]. Moreover, cultivating positive peer relationships at school—particularly prosocial friendships rooted in mutual support—serves as a pivotal mechanism to foster healthier behavioral patterns and enhance holistic well-being during adolescence.

Relocation for poverty alleviation has improved the previous community disadvantages to some extent, including the living environment, educational culture, and community atmosphere. From the perspective of social cognitive theory, improvements in community disadvantage can objectively increase access to health information and enhance access to health resources and can subjectively affect individual perceptions, including self-efficacy and outcome expectations for health, education, and social relationships, as well as a re-evaluation of life satisfaction through the development of personal cognition [57]. All of these factors may reduce depressive states directly or through increased life satisfaction and subjective well-being. This finding may contribute to the development of public policies to ameliorate depressive symptoms in adolescents, especially those whose families have lower socioeconomic status.

As mentioned earlier, the SCQ-AS scale lacks the family social capital dimension and fails to measure the intensity of family support (especially emotional support) and its impact on adolescents’ life satisfaction and depression among the relocated adolescents, whereas the importance of family support on adolescents’ mental health has been confirmed by many studies [58]. The extent to which adolescent social capital influences life satisfaction and depression may be stronger if it can be measured in the future using a more comprehensive scale that includes family social capital dimensions. It is worth noting that the parents of the respondents in this survey had low educational attainment, and some studies have shown that life satisfaction is unevenly distributed between adolescents with high and low socioeconomic status [59,60]. However, another possible outcome of low parental education is lower expectations for the outcomes of their children’s schooling. Depressive symptoms in adolescents were associated with their achievement goals [61]. Jiao [62] showed that a lighter school load and lower outcome preferences were protective factors for adolescents’ life satisfaction. Additionally, Moksnes found that life satisfaction partially mediated the association between school stress and depressive symptoms [63].

This study yields some policy insights as follows: 1. Education authorities and schools should prioritize organizing structured extracurricular activities to foster peer-to-peer and student–teacher bonds, thereby cultivating trust and cohesion among adolescents. 2. Local governments and community administrators must allocate targeted funding to expand psychosocial support programs, ensuring relocated youth receive tailored care while reinforcing their sense of trust and communal support within resettlement neighborhoods.

### Limitations

Our findings should be considered in the context of the following limitations: First, this study used the SCQ-AS to measure social capital, which is widely used in China. However, due to the lack of family or kinship dimensions in this instrument, this study fails to adequately validate and discuss the relationship between family support and depression among migrant adolescents. In the future, the research team will consider developing a more appropriate social capital scale for adolescents that includes family, school, and community levels. Second, since this study was based on a cross-sectional survey, the results of the mediation analysis could only explain the associations between the variables, rather than identifying causal pathways. In future studies, we will consider a longitudinal design to observe changes in social capital and its causal relationship with depression among adolescents after relocation. Third, the instruments used in this study, such as the SCQ-AS, SWLS, and Depression Scale, have all been validated in the general population of adolescents, but not all have been validated in the population of relocated adolescents, which is a potential limitation.

## 5. Conclusions

This study suggests that adolescents in relocated communities are overall more satisfied with their lives, but some are still experiencing some form of depression. Both social capital and life satisfaction have a positive impact on depression, and adolescents who are more satisfied with their lives are better able to cope with the negative influences that may be present in their lives, thus helping to reduce depressive symptoms.

Further, school social cohesion and school friendships are the most important factors in helping adolescents increase life satisfaction and combat depression. Therefore, appropriate educational initiatives and community investments are essential to increase adolescents’ social capital and life satisfaction and to reduce and prevent depression.

## Figures and Tables

**Table 1:** Characteristics of the study population and basic information.

Variables	Categories	n	%
Gender	Boy	315	49.9
	Girl	316	50.1
Age (years)	10–13	347	55.0
	14–16	153	24.2
	17–19	131	20.8
Only child	Yes	90	14.3
	No	541	85.7
Father’s education	Elementary school	225	35.66
	Middle school	307	48.65
	High school	69	10.94
	College and above	30	4.75
Mather’s education	Elementary school	205	32.49
	Middle school	312	49.45
	High school	78	12.36
	College and above	36	5.71
Smoking	Yes	17	2.7
	No	614	97.3
Alcohol	Yes	15	2.4
	No	616	97.6
Outdoor exercise	Yes	455	72.1
	No	176	27.9

**Table 2:** Descriptive statistics and correlation analysis of social capital with life satisfaction and depression.

	$\bar{x}$ ± S	Life Satisfaction	Depression
Social capital (total)	31.96 ± 3.666	0.363 *	−0.362 *
School cohesion	10.71 ± 1.732	0.366 *	−0.345 *
School friendship	8.39 ± 1.141	0.214 *	−0.302 *
Neighborhood social cohesion	5.15 ± 1.190	0.191 *	−0.228 *
Trust	7.71 ± 1.466	0.139 *	−0.086 *
Life satisfaction	23.21 ± 6.282	1	−0.398 *
Depression	4.03 ± 5.503	−0.398 *	1

Note: * *p* < 0.05.

**Table 3:** Regression analysis of the mediating effect of life satisfaction on the association between social capital and depression.

Variables		Step 1-c	Step 2-a	Step 3-b	Step 3-c′
Social capital (total)	β	−0.55 *	0.63 *	−0.16 *	−0.44 *
	R^2^	0.13	0.13	0.17	
	F	97.00 *	91.80 *	62.13 *	
	*p*	˂0.001	˂0.001	˂0.001	
School cohesion	β	−1.09 *	1.37 *	−0.17 *	−0.87 *
	R^2^	0.12	0.13	0.15	
	F	85.23 *	97.17 *	56.60 *	
	*p*	˂0.001	˂0.001	˂0.001	
School friendship	β	−1.45 *	1.22 *	−0.21 *	−1.20 *
	R^2^	0.09	0.05	0.15	
	F	63.32 *	30.08 *	54.45 *	
	*p*	˂0.001	˂0.001	˂0.001	
Neighborhood social cohesion	β	−1.05 *	1.04 *	−0.22 *	−0.82 *
	R^2^	0.05	0.04	0.12	
	F	34.39 *	23.75 *	42.12 *	
	*p*	˂0.001	˂0.001	˂0.001	
Trust	β	−0.32 *	0.62 *	−0.17 *	−0.24 *
	R^2^	0.01	0.02	0.09	
	F	4.66 *	12.42 *	31.02 *	
	*p*	0.0313	0.0005	˂0.001	

Note: * *p* < 0.05.

**Table 4:** Mediating effects of life satisfaction and their proportion of the total effect.

Variables	Mediating Effect	Bootstrap 95% CI		Proportion of the Total Effect
		Lower Limit	Upper Limit	
Social capital (total)	−0.1008	0.06	0.15	18.20%
School cohesion	−0.2329	0.13	0.34	21.37%
School friendship	−0.2562	0.13	0.38	17.67%
Neighborhood social cohesion	−0.2288	0.13	0.37	21.79%
Trust	−0.1054	0.06	0.25	32.94%

## Data Availability

The raw data supporting the conclusions of this article will be made available by the authors on request.

## Abbreviations

The following abbreviations are used in this manuscript:

DASS-21	Depression Anxiety Stress Scale
SCQ-AS	Social Capital Questionnaire for Adolescent Students
SWLS	Satisfaction with Life Scale
CI	Confidence Interval
